# The Mediating Role of Immune Cells in the Genetically Predicted Relationship between Gut Microbiota and Puerperal Sepsis: A Mendelian Randomization Study

**DOI:** 10.2174/0118715303415101251129044507

**Published:** 2026-01-15

**Authors:** Yihui Chai, Chunsong Gu, Zhiliang Fan, Qian Li, Chunyan Liu, Jianjun Niu, Jun Li, Xiang Pu

**Affiliations:** 1 *School of Basic Medicine, Guizhou University of Traditional Chinese Medicine, Guiyang, Guizhou, China; *; 2 *Department of Orthopaedics, The Second Affiliated Hospital of Guizhou University of Traditional Chinese Medicine, Guiyang, Guizhou, China; *; 3 Tujia Institute of Medicine, Dejiang County Hospital of Traditional Chinese Medicine, Dejiang, Guizhou, China;; 4 Resource Institute for Chinese & Ethnic Materia Medica, Guizhou University of Traditional Chinese Medicine, Guiyang, Guizhou, China

**Keywords:** Gut microbiota, puerperal sepsis, immune cells, organ dysfunction syndrome, biomarkers, single nucleotide polymorphisms

## Abstract

**Introduction:**

The causal relationship between gut microbiota and puerperal sepsis (PS) remains unclear, and there is a lack of in-depth research regarding the potential mediating role of immune cells in this context.

**Objective:**

This study aims to investigate the causal relationship between gut microbiota and PS using Mendelian randomization (MR) analysis and to assess the mediating effects of immune cells on the risk of PS onset through mediation analysis.

**Materials and Methods:**

We selected data from large-scale genome-wide association studies (GWAS) involving 473 gut microbiota species, 731 immune cell phenotypes, and PS datasets. Univariate MR (UVMR) analysis was employed to explore the causal relationship between gut microbiota and PS, with the primary statistical method being inverse variance weighting (IVW). Multiple statistical models were applied for sensitivity analysis to minimize the confounding effects of horizontal pleiotropy and heterogeneity. Subsequently, a two-step mediation MR analysis was conducted to evaluate whether immune cells mediate the relationship between gut microbiota and PS.

**Results and Discussion:**

Analysis using various statistical models indicated that 11 gut microbiota species (*e.g.*, *Azorhizobium*, *Bacillus velezensis*, *CAG-245 sp000435175*, *Lentimicrobiaceae*, and *Providencia*) exhibited a causal relationship with PS. Further reverse causal analysis between PS and gut microbiota ruled out the possibility of reverse causality. The two-step mediation MR analysis demonstrated that the percentage of IgD-CD27- B cells (10.26%) and CD62L- monocytes (17.29%) partially mediated the effect of *CAG-245 sp000435175* on PS risk.

**Conclusion:**

This study provides evidence of a causal relationship between the abundance of certain gut microbiota species and PS, while also revealing a potential mediating role of immune cells. These findings offer valuable theoretical insights into personalized treatment strategies and the development of novel diagnostic biomarkers for PS.

## 1 INTRODUCTION

Puerperal sepsis (PS) is a severe reproductive tract infection that occurs from the rupture of membranes or delivery up to 42 days postpartum. Its clinical manifestations include pelvic pain, elevated body temperature (≥38.5°C), foul-smelling vaginal discharge, and delayed uterine involution [1]. As one of the leading causes of maternal mortality globally, PS is particularly prevalent in resource-poor regions. According to the World Health Organization, 15% of maternal deaths are associated with PS. In low-income countries, the incidence of PS ranges from 0.1% to 10%, resulting in approximately 75,000 maternal deaths annually. Complications of PS include chronic pelvic inflammatory disease, infertility, disseminated intravascular coagulation, multiple organ dysfunction syndrome, and end-stage organ failure [2, 3].

Although significant progress has been made in the prevention and treatment of PS, its global incidence and related maternal mortality remain high, especially in developing countries. Statistics indicate that approximately 5% to 10% of pregnant women are affected by PS, which can lead to adverse neonatal outcomes, such as preterm birth, neonatal sepsis, and low Apgar scores [4, 5]. The increasing incidence of pregnancy-related complications highlights the importance of early recognition and treatment. International guidelines emphasize the importance of early antimicrobial therapy, fluid resuscitation, and organ protection in the management of PS. Multidisciplinary collaboration has been shown to improve disease outcomes and reduce mortality. Consequently, the identification of diagnostic biomarkers and effective therapeutic approaches remains a key focus of research [6].

The gut microbiota (GM) is often referred to as the “metabolic organ” due to its crucial role in host physiology, metabolism, immune regulation, and nutritional balance [7]. Host immune responses are modulated by interactions between single-layer epithelial cells and microbial antigens or metabolites, thereby influencing disease risk [8, 9]. Studies have shown that the abundance of specific gut bacteria correlates positively with the incidence of sepsis and 28-day survival rates [10]. Additionally, dysbiosis of the GM during pregnancy may exacerbate the pathogenesis of PS by reducing beneficial bacteria (*e.g.*, *Parabacteroides merdae*), thereby inducing macrophage necroptosis and inflammatory responses. Changes in the abundance of enterococci and Bacteroides species may also influence the severity of sepsis by modulating the immune response [11, 12]. While evidence supporting a close relationship between the GM and PS has been accumulating, the underlying mechanisms remain unclear.

Immune cells play a central role in defending the host against pathogens. PS is a form of sepsis characterized by an abnormal host immune response that involves significant disruption of both innate and adaptive immunity [13]. Various immune cell subsets exhibit significant dysfunction during different stages of PS. For example, monocytes with low expression of HLA-DR and high expression of the S100A gene contribute to immune suppression by inhibiting CD4+ T cell responses, while mature dendritic cells enriched with regulatory molecules promote the differentiation of CD4+ T cells into regulatory T cells [14, 15]. Furthermore, the activation of pathogen-associated molecular patterns and damage-associated molecular patterns triggers inflammatory cascades, resulting in a cytokine storm and systemic inflammatory response syndrome [16, 17]. During disease progression, T cell exhaustion, an increased proportion of regulatory T cells, and metabolic alterations in monocytes and macrophages further exacerbate immune dysfunction [18]. Studies have indicated that the expression of CD62L varies significantly in PS patients, suggesting its potential involvement in immune regulation during PS [19].

While a close association between GM and PS is widely accepted, the precise role of immune cells in this process remains unclear. Therefore, a deeper understanding of the interactions between GM, immune cells, and PS is crucial for elucidating the pathogenesis of this disease. Mendelian randomization (MR), a causal inference tool based on genetic variation, has increasingly been applied to investigate the causal relationship between GM and PS. This study leverages large-scale genomic data to analyze the causal relationship between GM and PS and further explores the potential role of immune cells in this process.

## 2 MATERIALS AND METHODS

### 2.1 Study Design

The experimental design of this study is illustrated in Fig. (**1**). Initially, we employed univariable Mendelian randomization (UVMR) to analyze the positive causal relationship between GM and the risk of PS. Subsequently, we treated PS as the exposure variable and explored its potential reverse causal effects on GM. To ensure the scientific rigor of the experimental design and the reliability of the results, we validated three key hypotheses using multiple methods. These included reducing pleiotropic interference and controlling heterogeneity to minimize the risk of violating the assumptions.

Additionally, Fig. (**1**) also demonstrates the mediation analysis flow based on the causal relationship between GM and PS, comprising two steps: First, the causal effect of GM on immune cells (β1, *p*<0.05) was assessed. Second, the causal effect of immune cells on PS (β2, *p*<0.05) was combined with the total effect of GM on PS (β, *p*<0.05) to compute both the mediation effect and the direct effect. The mediation effect was calculated as β1β2, the direct effect as β-β1β2, and the proportion of the mediation effect as β1β2/β [20].

### 2.2 Data Sources

#### 2.2.1 Gut Microbiota Data

The GM data used in this study were derived from a database provided by Qin *et al*. [21]. This dataset includes information from 5,959 genetically matched individuals, encompassing GM composition, dietary habits, and health records. The data were analyzed to investigate the influence of genetic variation on GM abundance. A total of 567 independent single nucleotide polymorphisms (SNPs) were identified as significantly associated with microbiota taxonomic units through a GWAS. These GWAS results provide critical insights into the genetic underpinnings of GM.

#### 2.2.2 Puerperal Sepsis Data

PS data were obtained from the FinnGen database, which integrates genomic and health data from nearly half a million Finnish individuals. By combining genetic information with clinical records, the database aims to explore associations between genetic variation and disease trajectories. In this study, the GWAS data for PS were sourced from the FinnGen project, with the phenocode labeled as “Puerperal Sepsis”. The dataset included a total of 375,082 samples, comprising 4,724 cases and 244,020 controls. Relevant data can be accessed *via* pheno O15_PUERP_SEPSIS.

#### 2.2.3 Immune Cell Data

The immune cell GWAS data used in this study were derived from an in-depth genetic analysis of peripheral blood immune cell samples from 3,757 European individuals. These samples were analyzed using flow cytometry, covering 731 distinct circulating immune cell phenotypes, including absolute cell counts (n=118), median fluorescence intensity of surface antigens (n=389), morphological parameters (n=32), and relative cell counts (n=192). The study focused on seven major types of circulating immune cells: dendritic cells, B cells, T cells, monocytes, myeloid cells, natural killer cells, and regulatory T cells.

Analysis of approximately 22 million genetic variants revealed 122 significantly independent association signals, spanning 459 cell traits and 70 loci, of which 53 signals overlapped with known disease-related signals, primarily from autoimmune diseases [22]. These findings provide insights into the complex genetic regulation of immune cell subtypes and offer potential drug targets for the precision treatment of autoimmune diseases. The relevant GWAS summary statistics are available through the GWAS Catalog at https://www.ebi.ac.uk/gwas/publications/32929287, with access numbers ranging from GCST0001391 to GCST0002121.

## 3 INSTRUMENTAL VARIABLE PROCESSING

In this study, GM was treated as the exposure variable, and PS was treated as the outcome variable. To ensure the stability of the data and the accuracy of the results, the following criteria were applied to the instrumental variables (IVs): (a) IVs associated with GM should meet a genome-wide significance threshold of P < 1 × 10^−5^; (b) To meet the assumptions of MR, linkage disequilibrium (LD) analysis was conducted based on the European 1000 Genomes Project, with the requirement that the R^2^ of the IVs be < 0.001 and the LD window be set to 10,000 kb; (c) To minimize the potential impact of allele frequencies on the causal relationship between GM and PS, we assessed the strength of the genetic variation used as IVs *via* the F-statistic. An F-statistic ≤ 10 is considered weak, potentially introducing bias into the analysis. In contrast, an F-statistic > 10 indicates strong IVs, and thus IVs with an F-statistic below 10 were excluded from further analysis. In reverse MR analysis, the IVs for PS were required to meet the following conditions: P < 5 × 10^−8^, R^2^ < 0.001, and LD = 10,000 kb. Again, IVs with an F-statistic below 10 were excluded (details of the IVs for GM are provided in Supplementary Material **S1**, and for PS in Supplementary Material **S2**). R^2^ represents the proportion of phenotypic variance explained by SNPs, and it is calculated as:


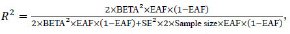



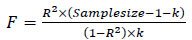


Where, k represents the number of SNPs included in the instrument.

For two-step mediation MR and multivariable MR (MVMR) analyses, the IVs for immune cells were required to meet the following conditions: P < 1 × 10^−5^, R^2^ < 0.1, and LD = 500 kb. IVs with an F-statistic below 10 were excluded (details of the IVs for immune cells are provided in Supplementary Material **S3**).

## 4 STATISTICAL METHODS

We collected the necessary data from public databases and conducted bidirectional MR analyses to explore the causal relationship between GM and PS. Immune cells were considered mediators, and a two-step mediation MR analysis was conducted to assess their influence on the genetic effects of GM on PS. MR analyses were performed in R (version 4.3.1) using the following packages: “TwoSampleMR” (version 0.5.7), “MendelianRandomization” (version 0.9.0), and “BWMR” (version 0.1.1).

### 4.1 UVMR Analysis: Core Effect Estimation and Preliminary Validation

UVMR analysis serves as the foundation for exploring the unidirectional causal relationship between the GM and PS. Its core lies in associating the exposure with the outcome through instrumental variables (SNPs), mainly achieving the dual objectives of “effect estimation + bias control” using the following methods [23]:

#### 4.1.1 IVW Method

It is considered the “gold standard” of MR analysis. It integrates the effect estimates of all instrumental variables to calculate the overall causal effect of the exposure on the outcome. It simultaneously evaluates the overall validity of the instruments (*i.e.*, whether they are strongly associated with the exposure and free from substantial pleiotropy), providing an initial reference for causal effect in subsequent analyses.

#### 4.1.2 Robust IVW and Penalized Robust IVW

Both are optimized extensions of IVW designed to reduce the influence of “outlier instruments” (*e.g.*, SNPs directly related to the outcome). Robust IVW down-weights extreme-effect instruments, while penalized robust IVW further penalizes outliers to avoid bias in the overall effect estimation.

#### 4.1.3 Contamination Mixture and Constrained Maximum Likelihood (cML–MA–BIC–DP)

Both aim to “actively identify and filter outliers.” Contamination mixture distinguishes “valid instruments” from “pleiotropic outliers” based on a probabilistic model, whereas cML–MA–BIC–DP excludes instruments violating MR assumptions using Bayesian Information Criterion and data-driven strategies, reducing bias at the source.

#### 4.1.4 MR-PRESSO and MR-Lasso

**Fig. (1):**
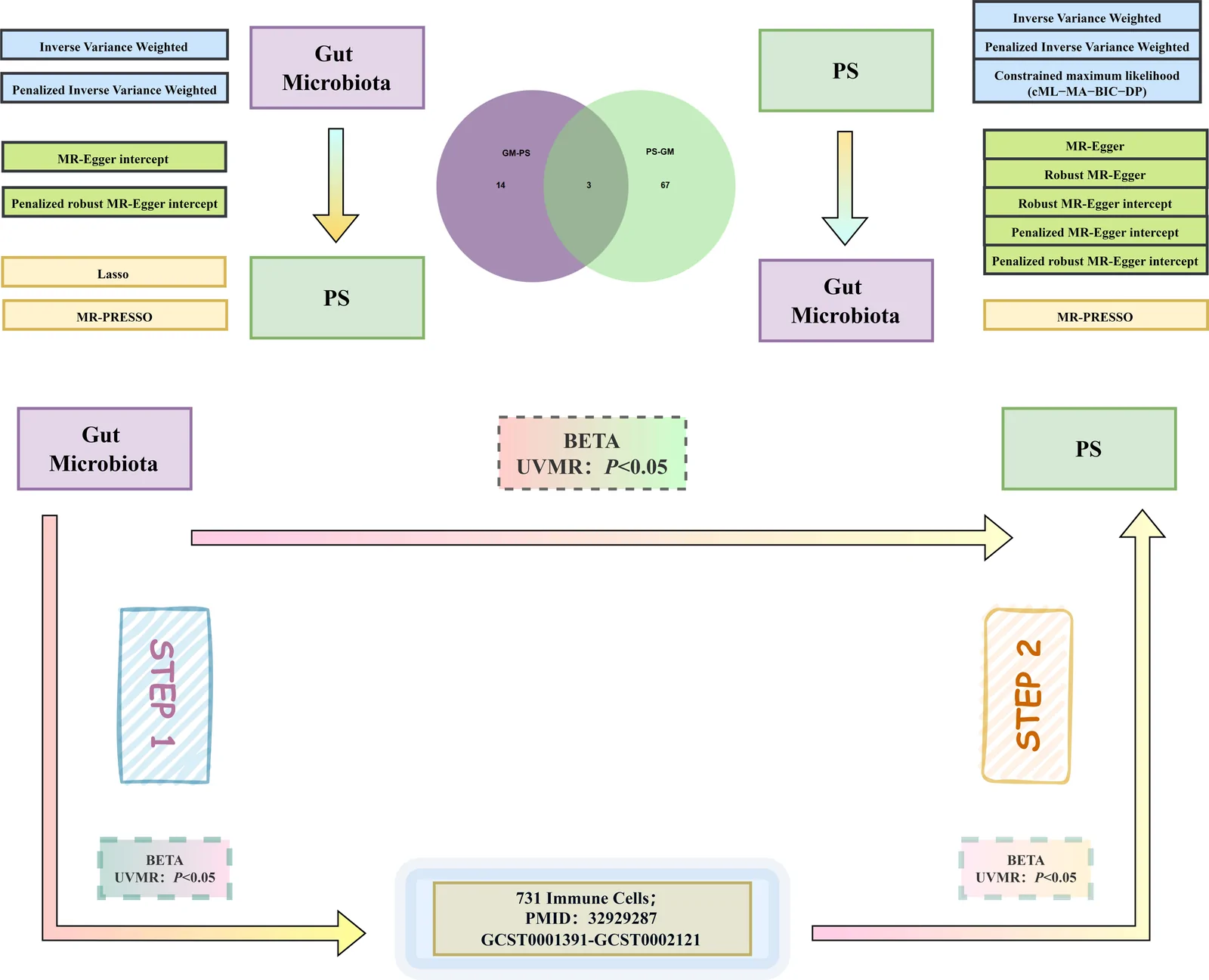
Flowchart of UVMR and MVMR analysis process.

These methods address “outlier correction and effect shrinkage”. MR-PRESSO quantifies the influence of outliers on effect estimates, recalculates effects after removing them, and verifies robustness; MR-Lasso applies L1 regularization to shrink the weights of weak instruments, minimizing false positives from weakly associated SNPs, thereby ensuring a more reliable causal effect of GM on PS (Fig. **1**).

### 4.2 Pleiotropy Testing: Validating MR Assumptions

Horizontal pleiotropy is a primary source of bias in MR. This study employs various MR-Egger–derived methods to achieve “pleiotropy testing + result correction”, with the following purposes:

#### 4.2.1 MR-Egger, Robust MR-Egger, and Penalized MR-Egger

These methods primarily test for “directional pleiotropy”; a significant regression intercept (*P* > 0.05 indicating no substantial pleiotropy) suggests no overall bias in instruments. Robust MR-Egger enhances resistance to outliers, while penalized MR-Egger penalizes extreme pleiotropic instruments, improving test accuracy.

#### 4.2.2 Intercept Test

It directly quantifies the degree of pleiotropy; a significant non-zero intercept indicates directional pleiotropy and requires further correction using other methods (*e.g.*, MR- PRESSO). An insignificant intercept suggests that instruments generally satisfy the MR assumptions, making causal effect estimates more reliable.

### 4.3 Two-Step Mediation MR Analysis: Elucidating the Mediating Role of Immune Cells

In the two-step mediation MR analysis, the first step involved the UVMR analysis of GM and immune cells, providing the effect estimate BETA1. The second step involved MVMR analysis of the positive mediators from the first step and GM, providing the effect estimate BETA2. In this process, the UVMR analysis between GM and PS provided the total effect BETA, and the mediation effect was calculated as BETA1 × BETA2, while the direct effect was calculated as BETA - BETA1 × BETA2. The proportion of the mediation effect was calculated as BETA1 × BETA2 / BETA. In the second step of the MVMR analysis, multivariable IVW was used to validate the IVs' effectiveness and to assess the weighted total effect using P-value evaluation.

## 5 RESULTS

### 5.1 Unidirectional Causal Effect of GM on Puerperal Sepsis PS

**Fig. (2):**
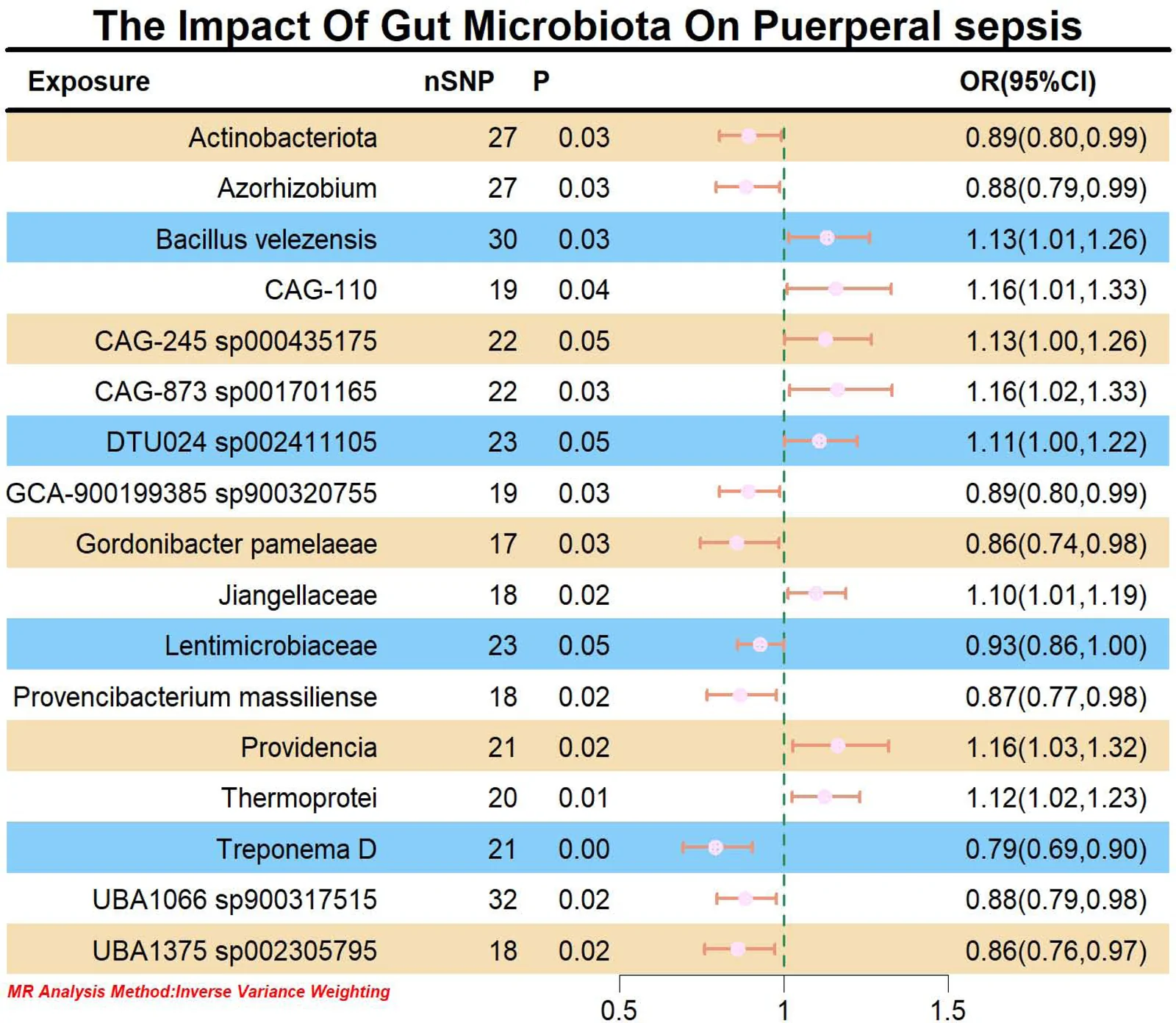
Forest plot of UVMR analysis for gut microbiota causal effect on PS.

**Fig. (3):**
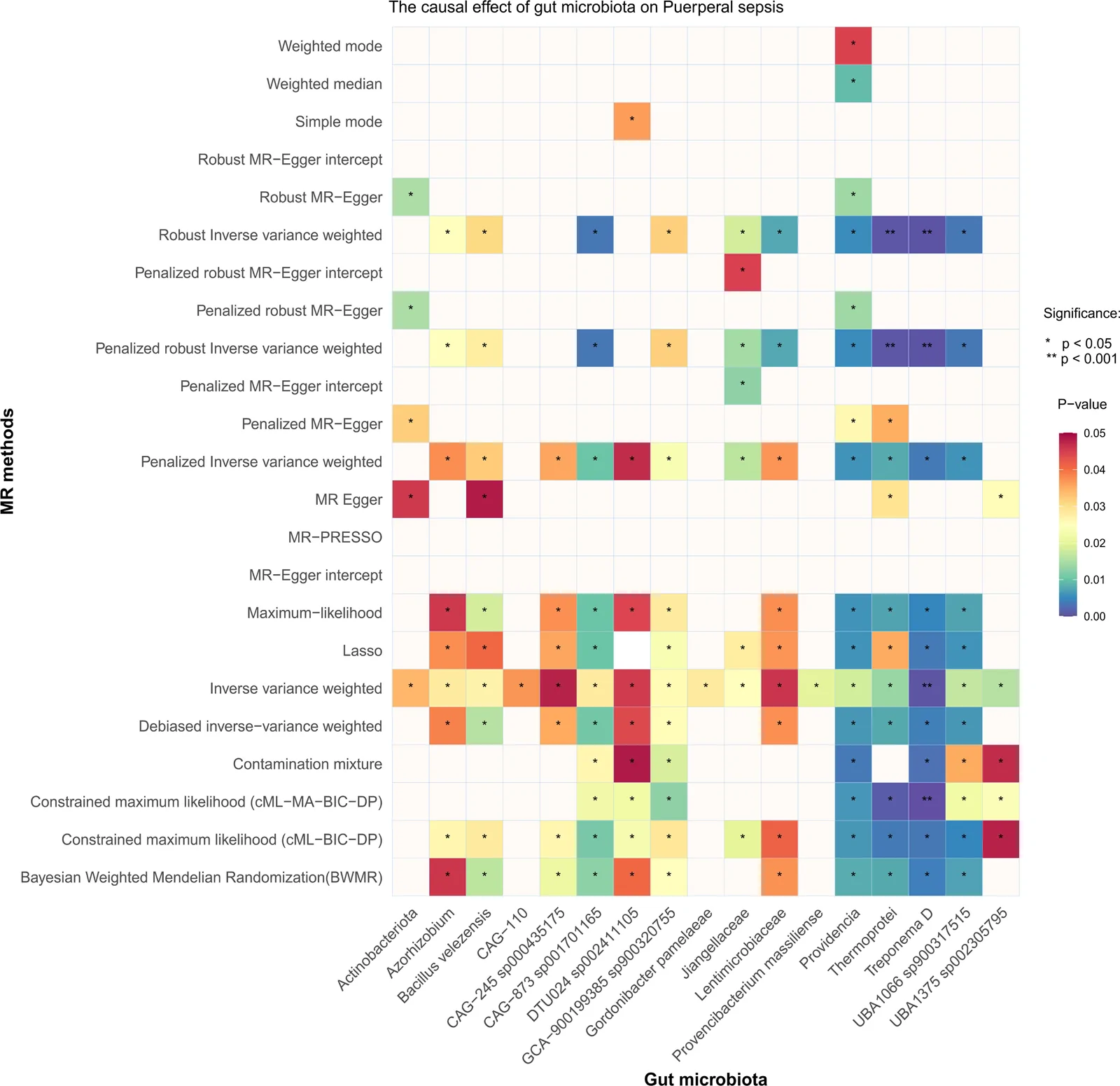
Heatmap of UVMR analysis for gut microbiota causal effect on PS.

Using the IVW method, we identified 24 GM species with significant causal relationships to PS (Fig. **2**, Supplementary Material **S4**). We then conducted robustness checks on these 17 results using methods, such as Robust IVW and Penalized Robust IVW, as well as various MR-Egger and intercept tests to minimize horizontal pleiotropy. To further eliminate outliers, we applied the MR-PRESSO method. Finally, MR-Lasso analysis was performed to enhance the robustness of the causal estimates from the IVW method (Fig. **3**, Supplementary Material **S5** and **6**). After rigorous filtering, we confirmed 11 GM species associated with PS, including: *Azorhizobium*, *Bacillus velezensis*, *CAG-245 sp000435175*, *CAG-873 sp001701165*, *DTU024 sp002411105*, *GCA-900199385 sp900320755*, *Lentimicrobiaceae*, *Providencia*, *Thermoprotei*, *Treponema D*, and *UBA1066 sp900317515*. Of these, 5 species were identified as protective factors, and 6 as risk factors.

### 5.2 Reverse Causal Effect of PS on GM

**Fig. (4):**
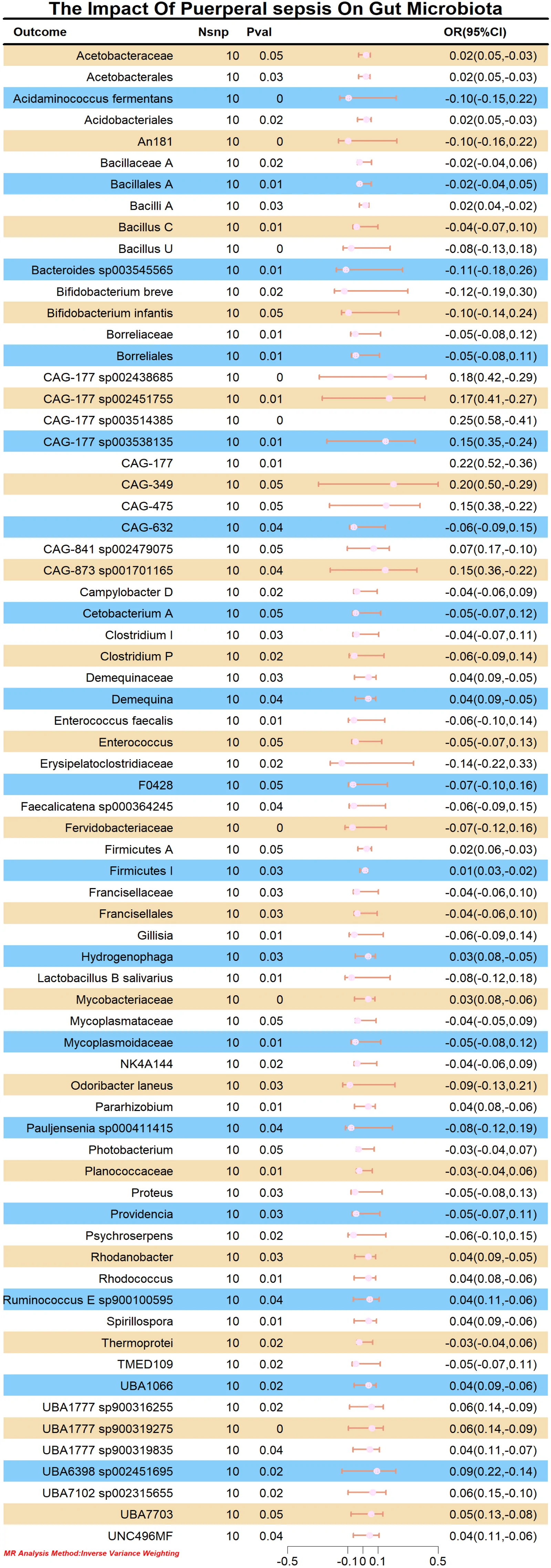
Forest Plot of UVMR analysis for PS causal effect on gut microbiota.

**Fig. (5):**
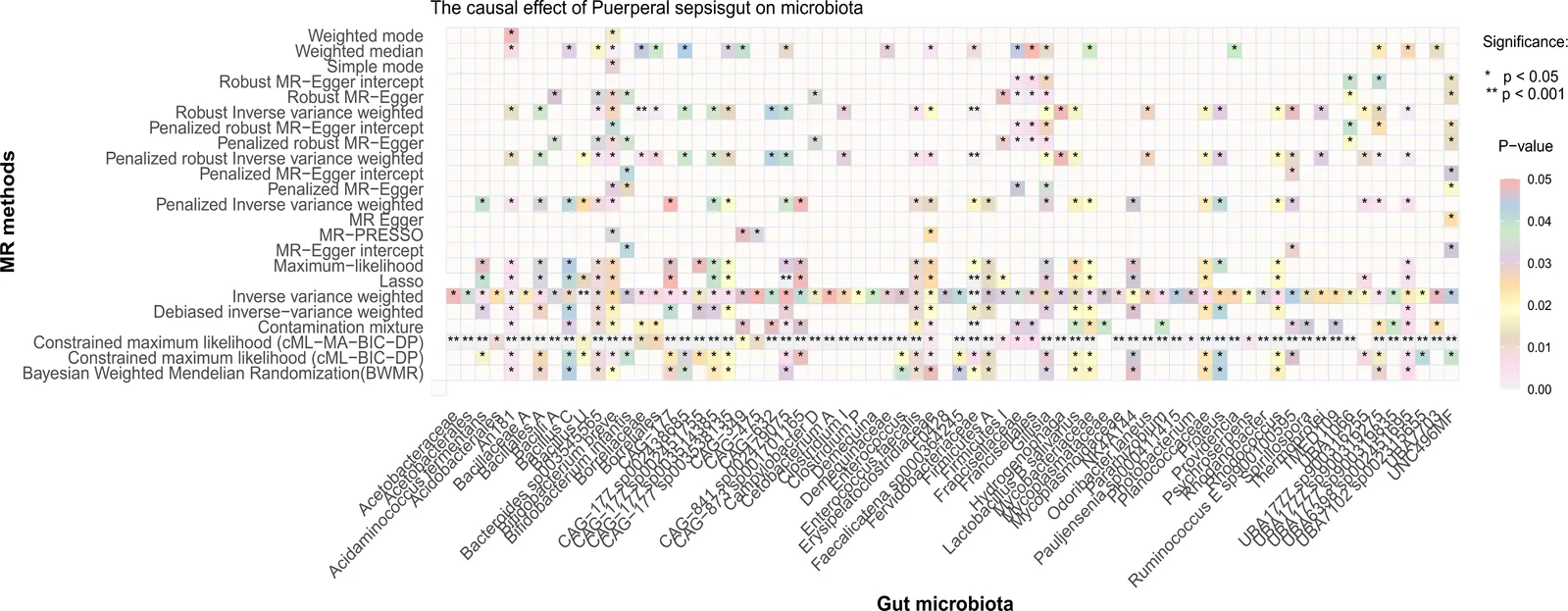
Heatmap of UVMR analysis for PS causal effect on gut microbiota.

Using the IVW method, we initially identified 70 GM species with a reverse causal relationship to PS (Fig. **4**, Supplementary Material **S7**). To improve the robustness of the results, we applied additional methods, such as Penalized IVW and cML−MA−BIC−DP, along with MR-Egger, Robust MR-Egger, Penalized MR-Egger Intercept, Robust MR-Egger Intercept, and Penalized Robust MR-Egger Intercept, to reduce the impact of directional pleiotropy. MR- PRESSO was also used to exclude outliers, further enhancing the robustness of the findings (Fig. **5**, Supplementary Material **S8** and **9**).

After rigorous filtering, we identified two GM species, *Bacteroides sp003545565* and *Campylobacter D*, that showed significant reverse causal effects on PS. To rule out reverse causality interference, we conducted an intersection analysis between these two species and the 11 positive species identified previously. The results showed no overlap, indicating that the forward causal relationships of the identified species were not influenced by reverse causality.

### 5.3 Mediating Role of Immune Cells in the Causal Relationship between GM and PS

To further explore the causal relationship between GM and PS, we incorporated 731 immune cell types into the UVMR analysis (Supplementary Material **S10**). We found that 49 GM-immune cell combinations had significant positive causal relationships, with key results shown in Fig. (**6**). The main causal effects are illustrated in Fig. (**7**), where an OR > 1 indicates that exposure is a risk factor, and an OR < 1 suggests that exposure is a protective factor (Supplementary Material **S11**). Further analysis revealed negative causal relationships between IgD^−^ CD27^−^ B cells (% of lymphocytes) and CD62L^−^ monocytes (% of monocytes) with PS (OR < 1). These findings provide important insights into the role of GM and immune cells in the pathogenesis of PS.

Moreover, in this study, *IgD-CD27-B cells* (10.26%) and CD62L-monocytes (17.29%) served as mediators, influencing the effect of *CAG-245 sp000435175* on PS risk (Supplementary Material **S12**).

## 6 DISCUSSION

PS is a life-threatening infectious disease that occurs after childbirth, primarily caused by obstetric infections, including postpartum and post-abortion infections, as well as associated organ dysfunction. PS is one of the leading causes of maternal mortality worldwide, accounting for 10.7% of maternal deaths. In high-income countries, the mortality rate from PS is well-controlled; however, in low-income regions, such as Sub-Saharan Africa, it remains a major cause of maternal deaths, with approximately 130,000 maternal deaths occurring annually [24]. Currently, the primary cause of PS is bacterial infection, and changes in the GM have been identified as a significant risk factor for the disease. Alterations in the GM can affect key antigens, such as the R28 protein, by interacting with host receptors like CEACAM1, thereby inhibiting tissue repair and immune responses and exacerbating infections [25].

The GM plays a crucial role in the pathogenesis of PS. Studies suggest that GM dysbiosis may influence the severity of PS by modulating the inflammatory response, and the composition of the microbiota directly impacts susceptibility to PS. Recent research has found that indole-3-propionic acid (IPA), a key metabolite, has neuroprotective properties by inhibiting NLRP3 inflammasome activation and IL-1β secretion, potentially alleviating neuroinflammatory responses induced by PS [26]. Additionally, secondary metabolites from the GM can enhance macrophage phagocytosis by activating the aryl hydrocarbon receptor, which helps control infections and reduce organ damage. This suggests that IPA may play a pivotal role in mitigating the multisystem damage caused by PS [27].

In this study, we identified 11 GM species associated with PS, including species from the phyla *Proteobacteria*, *Firmicutes*, and *Bacteroidetes*. Previous research has shown that *Proteobacteria* and *Firmicutes* remain dominant in the gut of critically ill PS patients [28], with the abundance of *Firmicutes* and *Proteobacteria* showing a dependency on disease severity [29]. *Firmicutes* play a dual role in sepsis. Increasing the proportion of beneficial bacteria, such as *Firmicutes,* can help regulate the GM-metabolic axis and improve intestinal barrier function, thus suppressing inflammation in the early stages of sepsis and reversing immunosuppression in the later stages [30]. However, in some cases, *Firmicutes* may also be associated with intestinal barrier dysfunction and inflammation, with its role varying depending on the bacterial strain and ecological environment [31]. By improving the ratio of *Firmicutes* to other microbiota (*e.g.*, *Bacteroidetes*), sepsis-induced lung injury and systemic inflammation can be alleviated, highlighting the importance of maintaining GM balance [32].

GM dysbiosis may be a key mechanism in the development of PS. The importance of *Firmicutes* in both neonatal and maternal microbiome development, especially in gut barrier function and immune regulation, suggests that modulating *Firmicutes* could be an effective intervention strategy. Studies have shown that a reduction in *Firmicutes* in PS patients may be linked to a decrease in GM diversity and the proliferation of harmful bacteria. Our findings indicate that species, such as *CAG-245 sp000435175,* are strongly causally related to PS. Although these species are widespread in European populations, there is a lack of research on their role in PS.

The increase in *Proteobacteria* abundance is closely linked to sepsis, particularly PS. Studies have shown that increased gut *Proteobacteria* prior to sepsis may create a conducive environment for pathogen colonization and could lead to bloodstream infections *via* intestinal translocation. *Providencia*, a representative genus of *Proteobacteria*, has gained attention in recent years for its pathogenic role in sepsis, particularly PS. *Providencia* activates the host's innate immune response through virulence factors, such as lipopolysaccharides and exotoxins, leading to the release of inflammatory mediators and immune dysregulation. Studies have reported that *Providencia* enhances NF-κB signaling, leading to increased cytokine expression, including IL-6 and TNF-α, which further contribute to the development of multiple organ dysfunction syndrome. Moreover, the increased antimicrobial resistance of *Providencia*, with genes, such as blaNDM-1 and blaOXA, complicates clinical treatment and limits the efficacy of standard antibiotic therapies [33]. Therefore, particular attention should be paid to the potential pathogenic role of *Providencia* in the microbiological diagnosis of puerperal infections. Personalized antimicrobial sensitivity testing should guide treatment plans to reduce PS mortality.

In this study, we systematically explored the causal relationships between 473 GM species and PS using large-scale genetic data and MR analysis. Additionally, we investigated the role of 731 circulating immune cell phenotypes in this process. To our knowledge, this is the first study to examine various circulating immune cell phenotypes as mediators of the genetic causal relationship between GM and PS. By applying strict inclusion criteria and sensitivity analyses, we identified 11 GM species significantly associated with PS, including *Azorhizobium*, *Bacillus velezensis*, *CAG-245 sp000435175*, *CAG-873 sp001701165*, *DTU024 sp002411105*, *GCA-900199385 sp900320755*, *Lentimicrobiaceae*, *Providencia*, *Thermoprotei*, *Treponema D*, and *UBA1066 sp900317515*. Among these, *CAG-245 sp000435175* demonstrated a causal relationship with PS, with an odds ratio (OR) of 1.16 (95% CI: 1.03, 1.32), suggesting that it is a risk factor for PS. Furthermore, our multivariable MVMR analysis revealed potential mediating mechanisms: a negative causal effect between *CAG-245 sp000435175* and the percentage of IgD-CD27-B cells and CD62L-monocytes in lymphocytes.

Immune cell dysfunction plays a crucial role in the immune response in sepsis, particularly in the interaction of memory B cells (CD27+ CD19+). CD27, a co-stimulatory molecule on lymphocytes, regulates the function and survival of T cells, B cells, and plasma cells [34]. Studies have shown that CD27+ B cells increase in sepsis, with elevated surface PD-1/PD-L1 expression, suggesting these cells may be in an exhausted state, impairing immune tolerance and reducing the ability to clear infections [35]. This suggests that the functional state of memory B cells may be pivotal in immune responses to sepsis. Notably, immune cell dysfunction is more pronounced in PS, where immune suppression in patients leads to loss of function of CD27+ B cells and T cells, potentially due to prolonged immune activation or dysregulation of immune tolerance, exacerbating the disease's complexity [36]. In patients with severe sepsis, the expression of co-stimulatory molecules, such as CD27, is closely linked to clinical outcomes. For example, in critically ill PS patients, CD27 and OX40 expression is increased on CD4+ T cells, while CD27 expression is reduced in CD4+CD27-CD28- T cells, with lower CD27 expression observed in non-surviving patients [37]. The imbalance of these co-stimulatory molecules likely contributes to immune dysfunction and increases mortality.

CD62L is a major adhesion molecule expressed on leukocytes that is involved in leukocyte activation, migration, and the regulation of inflammatory responses. In sepsis, the changes in CD62L-monocytes are closely associated with inflammation and immune escape. Low expression of CD62L on monocytes is significantly correlated with the onset of secondary sepsis. In critically ill patients, the absence of CD62L impairs monocyte migration during local inflammation and exacerbates systemic immune responses, potentially leading to tissue damage and organ dysfunction [38]. In PS, a severe postpartum infection, the immune system is often dysregulated due to the activation state during pregnancy, making women more susceptible to immune response disturbances, further promoting the development of PS. Low CD62L expression on monocytes may worsen the immune response to infection and negatively impact prognosis [39, 40]. Therefore, CD62L- monocytes should not be overlooked in sepsis, particularly in PS, where their role in altering immune function and inflammation can accelerate disease progression.

PS is a life-threatening postpartum infectious disease and a major cause of maternal mortality worldwide. Its main causes include postpartum infections, post-abortion infections, and GM dysbiosis, with the latter recognized as a significant risk factor. Changes in GM regulate immune and inflammatory responses, thus influencing the severity of the disease. Opportunistic pathogens, such as *Providencia* and CAG-245 sp000435175, have attracted increasing attention in PS pathogenesis due to their multidrug resistance, complicating treatment. Our study, through genetic data and MR analysis, reveals causal relationships between GM and PS, highlighting the association between *Providencia*, *CAG-245 sp000435175*, and immune cell dysfunction, particularly in IgD-CD27-B cells and CD62L-monocytes. In conclusion, immune cell function plays a pivotal role in mediating GM's influence on PS and may serve as potential biomarkers for predicting and intervening in PS.

The GM and host immune system form a highly coordinated crosstalk network, which plays a key role in the development of PS. In postpartum women, GM is prone to dysbiosis due to childbirth-related trauma, hormonal fluctuations, and immune adjustments, characterized by a reduction of beneficial bacteria and overgrowth of pathogenic species. This dysbiosis influences systemic inflammatory responses through three main pathways: (1) metabolite-mediated immune regulation, such as short-chain fatty acids, including propionate and butyrate, which inhibit glycolysis in monocytes, reduce IL-6 and TNF-α production, and promote regulatory T cell differentiation *via* GPR43/41 signaling [41, 42]; (2) pathogenic bacterial metabolites activating immune signaling pathways, for instance, *Desulfovibrio vulgaris* induces MAPK and NF-κB activation in macrophages, increasing IL-1β and IL-8 secretion [43]; and (3) impaired intestinal barrier function allowing bacterial components, such as LPS, to enter the circulation, activate TLR4/MyD88/NF-κB signaling, and induce systemic inflammation, creating a “barrier damage–microbiota translocation–inflammation amplification” loop [44-46]. Additionally, microbial metabolites, such as IPA, act *via* the aryl hydrocarbon receptor to inhibit epithelial apoptosis and attenuate inflammation; the abundance of IPA-producing bacteria decreases in PS, weakening this protective effect [47, 48]. Postpartum hyperglycemia and hormonal fluctuations may further exacerbate microbiota imbalance and immune dysregulation, promoting pathogen translocation and inflammatory mediator release [49, 50]. Probiotic interventions can remodel the GM, increase beneficial bacterial abundance, suppress TLR4 and NF-κB activation, and enhance IL-10 secretion, thereby alleviating systemic inflammation [45, 51]. In summary, the GM modulates inflammatory mediators and systemic immune responses through bidirectional metabolic and signaling pathways, constituting a critical pathological mechanism in PS. Our MR study further confirms that specific gut microbial species, such as CAG-245 sp000435175, causally influence PS susceptibility by regulating immune cell functions, including IgD^−^ CD27^−^ B cells and CD62L^−^ monocytes, supporting the central role of microbiota-immune crosstalk in PS.

Our MR study has both strengths and limitations. First, the GM database used in this study is based on metagenomic sequencing, which provides higher resolution than 16S rRNA gene sequencing, allowing for finer classification units (such as genus or phylum). We also performed sensitivity and heterogeneity analyses using multiple statistical models to account for different pleiotropic patterns, enhancing the robustness of our results. However, there are some limitations. A key limitation of this study is that the genetic instruments for the GM were derived from GWAS data based on the general population, which included men and women without a history of childbirth, making them not fully representative of the specific population affected by PS (postpartum women). Although we applied multiple sensitivity analyses to minimize potential biases, this mismatch may still affect the accuracy of our results. Ideally, future studies should utilize GM GWAS data from women with a history of childbirth, which would represent an important direction for further research. GM is influenced by various environmental factors, such as diet, lifestyle, and medication. While the MR method reduces confounding factors through genetic variation, it cannot fully eliminate gene-environment interactions (*e.g.*, dietary habits, lifestyle, and climate). Furthermore, PS has a rapid onset and progression, so there may be some limitations in using genetic variation to analyze the disease. Additionally, our study population was derived from the FinnGen database, with most data from European ancestry. Given the differences in diet, metabolites, and microbiota across regions and ethnicities, caution is needed when generalizing these findings to other populations. Future studies should include more diverse populations to enhance the generalizability of the results.

## CONCLUSION

This study comprehensively investigates the causal relationships between gut microbiota, immune cells, and puerperal sepsis, revealing several positive and negative causal effects as well as bidirectional relationships. Our findings suggest that immune cells mediate the relationship between gut microbiota and PS, providing mechanistic insights that may guide future research on microbiome-based interventions and potential diagnostic approaches.

## Figures and Tables

**Fig. (6):**
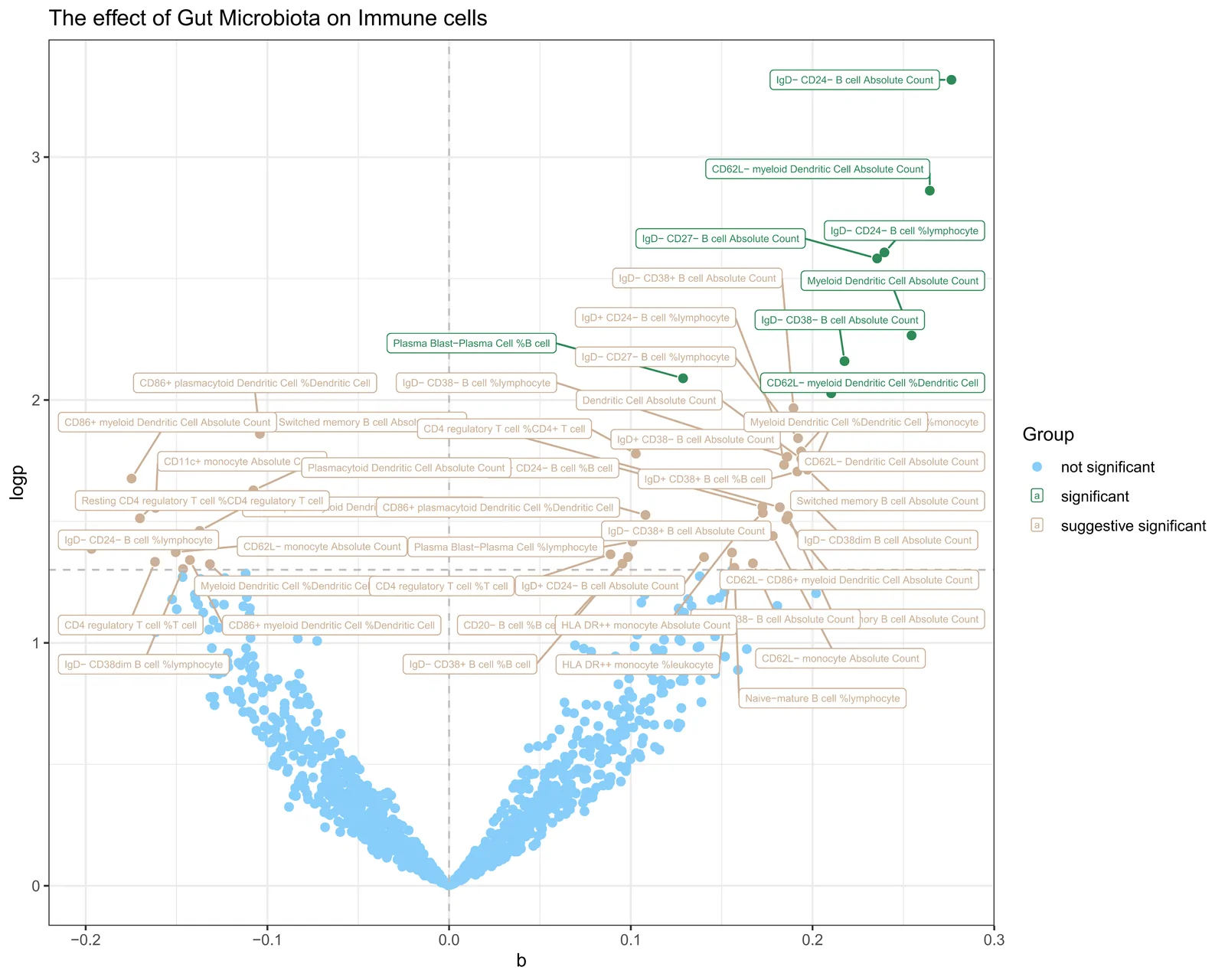
Volcano plot of UVMR analysis for major gut microbiota causal effect on immune cells (mediators).

**Fig. (7):**
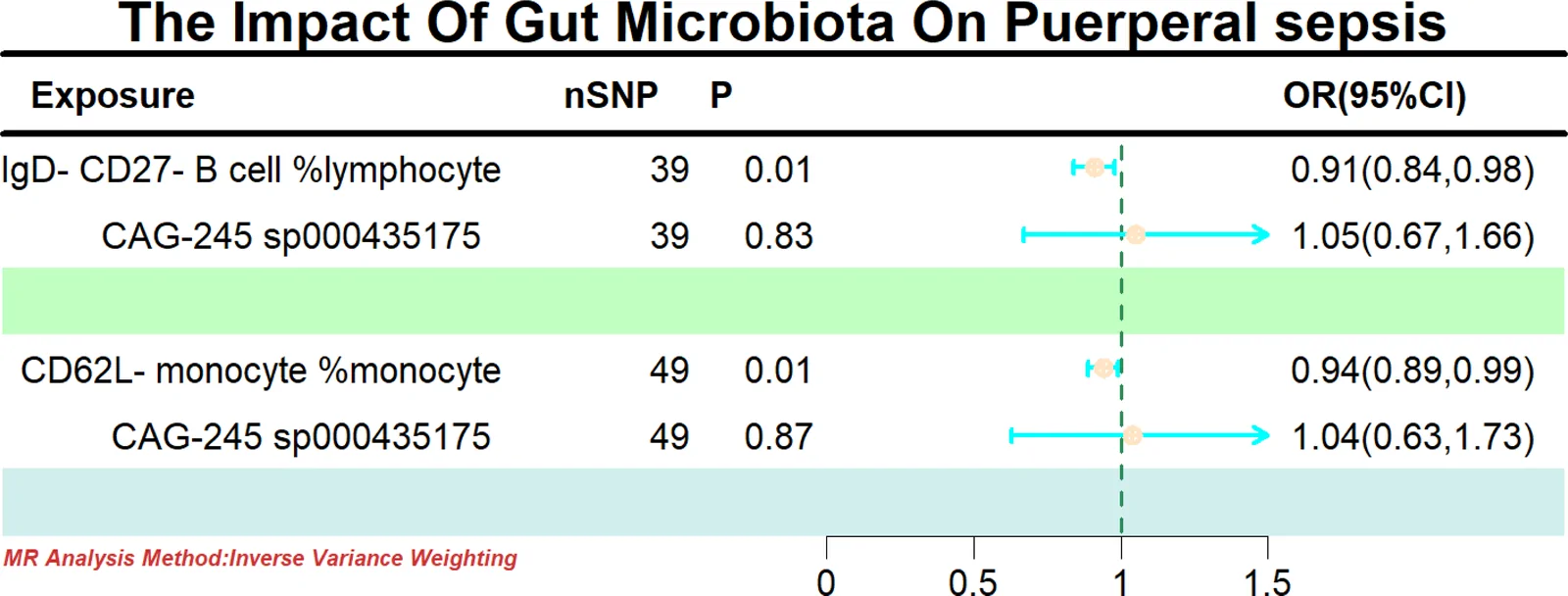
Forest plot of MVMR analysis for positive immune cell mediators in the causal effect of gut microbiota on PS.

## Data Availability

The raw data of proteomics and quantitative analysis in the current study are available from the corresponding author upon reasonable request.

## LIST OF ABBREVIATIONS

PS: Puerperal Sepsis
GWAS: Genome-wide Association Studies
IVW: Inverse Variance Weighting
GM: Gut Microbiota
MR: Mendelian Randomization
LD: Linkage Disequilibrium
UVMR: Univariable Mendelian Randomization
IVs: Instrumental Variables
MVMR: Multivariable Mendelian Randomization
cML–MA–BIC–DP: Contamination Mixture and Constrained Maximum Likelihood
IPA: Indole-3-Propionic Acid
OR: Odds Ratio

## STANDARDS OF REPORTING

STROBE-MR guidelines were followed.
