# Supplementary material for: The Mediating Role of Immune Cells in the Genetically Predicted Relationship between Gut Microbiota and Puerperal Sepsis: A Mendelian Randomization Study
Source: Endocr Metab Immune Disord Drug Targets. 2026 Jan 15;26:E18715303415101. doi: 10.2174/0118715303415101251129044507 (PMC13531649; doi:10.2174/0118715303415101251129044507)
Supplement: Supplementary file 1 [file EMIDDT-26-E18715303415101_SD1.pdf]

Supplementary Material

The Mediating Role of Immune Cells in the Genetically Predicted Relationship between Gut Microbiota and Puerperal Sepsis: A Mendelian Randomization Study

Yihui Chai[1,†], Chunsong Gu[2,†], Zhiliang Fan[1], Qian Li[1], Chunyan Liu[3], Jianjun Niu[3], Jun Li[4] and Xiang Pu[1,*]

[1]School of Basic Medicine, Guizhou University of Traditional Chinese Medicine, Guiyang, Guizhou, China [2]Department of Orthopaedics, The Second Affiliated Hospital of Guizhou University of Traditional Chinese Medicine, Guiyang, Guizhou, China; [3]Tujia Institute of Medicine, Dejiang County Hospital of Traditional Chinese Medicine, Dejiang, Guizhou, China; [4]Resource Institute for Chinese & Ethnic Materia Medica, Guizhou University of Traditional Chinese Medicine, Guiyang, Guizhou, China

STROBE-MR checklist of recommended items to address in reports of Mendelian randomization studies[1 2]

Item No.	Section	Checklist item	Page No.	Relevant text from manuscript
1	**TITLE and ABSTRACT**	Indicate Mendelian randomization (MR) as the study's design in the title and/or the abstract if that is a main purpose of the study	1	"A Mendelian Randomization Study" appears in the title; the abstract states "We performed a two-sample Mendelian randomization (MR) study…"
	**INTRODUCTION**			
2	**Background**	Explain the scientific background and rationale for the reported study. What is the exposure? Is a potential causal relationship between exposure and outcome plausible? Justify why MR is a helpful method to address the study question	2-3	"The gut microbiota (GM) is often referred to as the 'metabolic organ'... PS is a form of sepsis characterized by an abnormal host immune response... MR, a causal inference tool based on genetic variation, has increasingly been applied to investigate the causal relationship between gut microbiota and PS."
3	**Objectives**	State specific objectives clearly, including pre-specified causal hypotheses (if any). State that MR is a method that, under specific assumptions, intends to estimate causal effects	3	"This study leverages large-scale genomic data to analyze the causal relationship between gut microbiota and PS and further explores the potential role of immune cells in this process."
	**METHODS**			
4	**Study design and data sources**	Present key elements of the study design early in the article. Consider including a table listing sources of data for all phases of the study. For each data source contributing to the analysis, describe the following:	4-5	
	a)	Setting: Describe the study design and the underlying population, if possible. Describe the setting, locations, and relevant dates, including periods of recruitment,	4	"We performed a two-sample MR study using GWAS summary statistics...

		exposure, follow-up, and data collection, when available.		datasets included 473 gut microbiota species, 731 immune cell phenotypes, and PS data.”
	b)	Participants: Give the eligibility criteria, and the sources and methods of selection of participants. Report the sample size, and whether any power or sample size calculations were carried out prior to the main analysis	4-5	“Gut microbiota: 5,959 individuals from Youwen Qin et al. Immune cells: 3,757 European individuals... PS: FinnGen database with 375,082 samples (4,724 cases, 244,020 controls).”
	c)	Describe measurement, quality control and selection of genetic variants	5	“IVs selected with genome-wide significance ($P < 1\times10^{-5}$), LD R^2<0.001, F-stat >10...”
	d)	For each exposure, outcome, and other relevant variables, describe methods of assessment and diagnostic criteria for diseases	5	“Gut microbiota GWAS from metagenomic data... PS defined by FinnGen phenocode O15_PUERP_SEPSIS.”
	e)	Provide details of ethics committee approval and participant informed consent, if relevant	27	“All summary statistics... obtained from public databases and published studies. The original studies received ethical approval and obtained individual consent.”
5	**Assumptions**	Explicitly state the three core IV assumptions for the main analysis (relevance, independence and exclusion restriction) as well assumptions for any additional or sensitivity analysis	5	“To ensure the scientific rigor... we validated three key hypotheses... included reducing pleiotropic interference and controlling heterogeneity.”
6	**Statistical methods: main analysis**	Describe statistical methods and statistics used		
	a)	Describe how quantitative variables were handled in the analyses (i.e., scale, units, model)	6	“Effect estimates were expressed as ORs with 95% CIs; univariate and multivariable IVW applied.”
	b)	Describe how genetic variants were handled in the analyses and, if applicable, how their weights were selected	5-6	“Variants with genome-wide significance; LD pruning (R^2<0.001); F-statistic used for strength assessment.”
	c)	Describe the MR estimator (e.g. two-stage least squares, Wald ratio) and related statistics. Detail the included covariates and, in case of two-sample MR, whether the same covariate set was used for adjustment in the two samples	6	“Inverse variance weighted (IVW) used as primary MR estimator; also MR-Egger, MR-PRESSO, MR-Lasso.”
	d)	Explain how missing data were addressed		Not applicable
	e)	If applicable, indicate how multiple testing was addressed	6	“Robustness checks with MR-Egger, Penalized IVW, cML−MA−BIC−DP; P-value thresholds and pleiot-

				ropy filtering."
7	**Assessment of assumptions**	Describe any methods or prior knowledge used to assess the assumptions or justify their validity	6-7	"Various MR-Egger and intercept tests... were used to test for directional pleiotropy... P > 0.05 considered no directional pleiotropy."
8	**Sensitivity analyses and additional analyses**	Describe any sensitivity analyses or additional analyses performed (e.g. comparison of effect estimates from different approaches, independent replication, bias analytic techniques, validation of instruments, simulations)	6-7	"MR-PRESSO and MR-Lasso... Penalized MR-Egger... Contamination Mixture... Constrained Maximum Likelihood..."
9	**Software and pre-registration**			
	a)	Name statistical software and package(s), including version and settings used	6	"R version 4.3.1; 'TwoSampleMR' (v0.5.7), 'MendelianRandomization' (v0.9.0), 'BWMR' (v0.1.1)."
	b)	State whether the study protocol and details were pre-registered (as well as when and where)		Not applicable
	**RESULTS**			
10	**Descriptive data**			
	a)	Report the numbers of individuals at each stage of included studies and reasons for exclusion. Consider use of a flow diagram	4-5	"Gut microbiota: 5,959; Immune cells: 3,757; PS: 375,082... Exclusion based on F-stat < 10 and LD filtering."
	b)	Report summary statistics for phenotypic exposure(s), outcome(s), and other relevant variables (e.g. means, SDs, proportions)	5	SNP significance levels ($P < 1\times10^{-5}$); effect estimates (ORs with CIs) reported in Results.
	c)	If the data sources include meta-analyses of previous studies, provide the assessments of heterogeneity across these studies	6-7	"MR-Egger, IVW, Q statistics used to assess heterogeneity... MR-PRESSO used to filter outliers."
	d)	For two-sample MR:<br><br>i. Provide justification of the similarity of the genetic variant-exposure associations between the exposure and outcome samples<br><br>ii. Provide information on the number of individuals who overlap between the exposure and outcome studies	5	All samples of European ancestry (e.g., FinnGen and GWAS Catalog); harmonized using 1000 Genomes LD panel.
11	**Main results**			
	a)	Report the associations between genetic variant and exposure, and between genetic variant and outcome, preferably on an interpretable scale	7-8	"Identified 24 gut microbiota species... 11 after filtering... including ORs (e.g., OR=1.16, 95% CI: 1.03–1.32)"
	b)	Report MR estimates of the relationship between exposure and outcome, and the measures of uncertainty from the MR analysis, on an interpretable scale, such as	7-8	"Causal estimates (e.g., OR > 1 or < 1)... CI reported... Mediation effect calculated

		odds ratio or relative risk per SD difference		as β1×β2.”
	c)	If relevant, consider translating estimates of relative risk into absolute risk for a meaningful time period		Not applicable
	d)	Consider plots to visualize results (e.g. forest plot, scatterplot of associations between genetic variants and outcome versus between genetic variants and exposure)	28–29	“Figures 2–7 include forest plots, heatmaps, volcano plots...”
12	**Assessment of assumptions**			
	a)	Report the assessment of the validity of the assumptions	6–7	“No significant pleiotropy (Egger intercept P > 0.05); F-stat > 10; LD < 0.001.”
	b)	Report any additional statistics (e.g., assessments of heterogeneity across genetic variants, such as I^2, Q statistic or E-value)	6–7	“Heterogeneity addressed using Q statistic, cML−MA−BIC−DP... MR-Egger...”
13	**Sensitivity analyses and additional analyses**			
	a)	Report any sensitivity analyses to assess the robustness of the main results to violations of the assumptions	6–7	“Robust IVW, Penalized IVW, MR-PRESSO, MR-Lasso used.”
	b)	Report results from other sensitivity analyses or additional analyses	7-8	“Bidirectional MR and mediation analysis reported... Two-step MR for immune cells.”
	c)	Report any assessment of direction of causal relationship (e.g., bidirectional MR)	7	“Reverse MR identified 2 species... no overlap with forward results; reverse causality ruled out.”
	d)	When relevant, report and compare with estimates from non-MR analyses		Not applicable
	e)	Consider additional plots to visualize results (e.g., leave-one-out analyses)	Supplementary	“Figures and Supplementary Figures include heatmaps and forest plots; leave-one-out not specifically shown.”
	**DISCUSSION**			
14	**Key results**	Summarize key results with reference to study objectives	9–10	“Identified 11 species... 2 immune cell types as mediators... CAG-245 sp000435175 causally linked to PS.”
15	**Limitations**	Discuss limitations of the study, taking into account the validity of the IV assumptions, other sources of potential bias, and imprecision. Discuss both direction and magnitude of any potential bias and any efforts to address them	11	“Environmental factors... gene-environment interaction... data limited to European ancestry... not generalizable.”
16	**Interpretation**			
	a)	Meaning: Give a cautious overall interpretation of results in the context of their limitations and in compari-	10-11	“Immune cells mediate gut microbiota’s effect on PS... may inform interventions

		son with other studies		and biomarker development."
	b)	Mechanism: Discuss underlying biological mechanisms that could drive a potential causal relationship between the investigated exposure and the outcome, and whether the gene-environment equivalence assumption is reasonable. Use causal language carefully, clarifying that IV estimates may provide causal effects only under certain assumptions	9-11	"Discussed $CD62L^-$ monocytes, $CD27^-$ B cells... NF-κB, cytokine responses..."
	c)	Clinical relevance: Discuss whether the results have clinical or public policy relevance, and to what extent they inform effect sizes of possible interventions	10-11	"May serve as biomarkers... suggests personalized treatment strategies..."
17	**Generalizability**	Discuss the generalizability of the study results (a) to other populations, (b) across other exposure periods/timings, and (c) across other levels of exposure	11	"Most data from Europeans; generalizability to other populations needs further validation."
	**OTHER INFORMATION**			
18	**Funding**	Describe sources of funding and the role of funders in the present study and, if applicable, sources of funding for the databases and original study or studies on which the present study is based	26	"Funding: Guizhou Science and Technology Department, National Natural Science Foundation of China..."
19	**Data and data sharing**	Provide the data used to perform all analyses or report where and how the data can be accessed, and reference these sources in the article. Provide the statistical code needed to reproduce the results in the article, or report whether the code is publicly accessible and if so, where	26	"Raw data available from corresponding author upon request; public summary data used."
20	**Conflicts of Interest**	All authors should declare all potential conflicts of interest	26	"No potential conflict of interest was reported by the author(s)."

This checklist is copyrighted by the Equator Network under the Creative Commons Attribution 3.0 Unported (CC BY 3.0) license.

1. Skrivankova VW, Richmond RC, Woolf BAR, Yarmolinsky J, Davies NM, Swanson SA, et al. Strengthening the Reporting of Observational Studies in Epidemiology using Mendelian Randomization (STROBE-MR) Statement. JAMA. 2021;under review.

2. Skrivankova VW, Richmond RC, Woolf BAR, Davies NM, Swanson SA, VanderWeele TJ, et al. Strengthening the Reporting of Observational Studies in Epidemiology using Mendelian Randomisation (STROBE-MR): Explanation and Elaboration. BMJ. 2021;375:n2233.
